# Paving the Way Towards Universal Chimeric Antigen Receptor Therapy in Cancer Treatment: Current Landscape and Progress

**DOI:** 10.3389/fimmu.2020.604915

**Published:** 2020-12-10

**Authors:** Yixi Zhang, Pan Li, Hongyu Fang, Guocan Wang, Xun Zeng

**Affiliations:** State Key Laboratory for Diagnosis and Treatment of Infectious Diseases, National Clinical Research Center for Infectious Diseases, Collaborative Innovation Center for Diagnosis and Treatment of Infectious Diseases, The First Affiliated Hospital, School of Medicine, Zhejiang University, Hangzhou, China

**Keywords:** chimeric antigen receptor, graft-*versus*-host disease, host-*versus*-graft activities, adoptive cell therapy, cancer immunotherapy

## Abstract

Chimeric antigen receptor (CAR) therapy has been proved effective in a stream of clinical trials, especially in hematologic malignancies. However, current CAR therapy is highly personalized as cells used are derived from patients themselves, which can be costly, time-consuming, and sometimes fails to achieve optimal therapeutic results due to poor quality/quantity of patient-derived cells. On the contrary, universal CAR therapy, which is based on healthy individuals’ cells, circumvents several limitations of current autologous CAR therapy. To achieve the universality of CAR therapy, the allogeneic cell transplantation related issues, such as graft-*versus*-host disease (GVHD) and host-*versus*-graft activities (HVGA), must be addressed. In this review, we focus on current progress regarding GVHD and HVGA in the universal CAR therapy, followed by a universal CAR design that may be applied to allogeneic cells and a summary of key clinical trials in this field. This review may provide valuable insights into the future design of universal CAR products.

## Introduction

Cancer immunotherapy aims at triggering and augmenting immunity in cancer settings. Currently, there are two effective modalities of immunotherapy: monoclonal antibody (mAb) therapy and adoptive cell therapy (ACT). The success of autologous tumor-infiltrating lymphocytes (TILs) in the metastatic melanoma (1) and relapsed leukemia (2) in the 1980s heralded the era of ACT. In the past decades, scientists have shown increasing appreciation towards this field, as several phase III clinical trials have consistently shown improvement in the overall survival rate in advanced-stage cancers (3–6). More importantly, recent technical advances (e.g. cellular engineering and *ex vivo* cell manufacturing) have broadened the scope of ACT applications and enhanced the tumor-specific immune response in cancer treatments. As an extremely important type of ACT, CAR therapy has been perceived as a major breakthrough in cancer treatment and gained commercial approval by U.S. Food and Drug Administration, including Kymriah (tisagenlecleucel) **(** 7 **)** for relapse or refractory acute lymphoblastic leukemia in 2017, Yescarta (axicabtagene ciloleucel) (8) for certain types of large B cell lymphoma in 2017, and Tecartus (brexucabtagene autoleucel) for mantle cell lymphoma in 2020.

**Figure 1:**
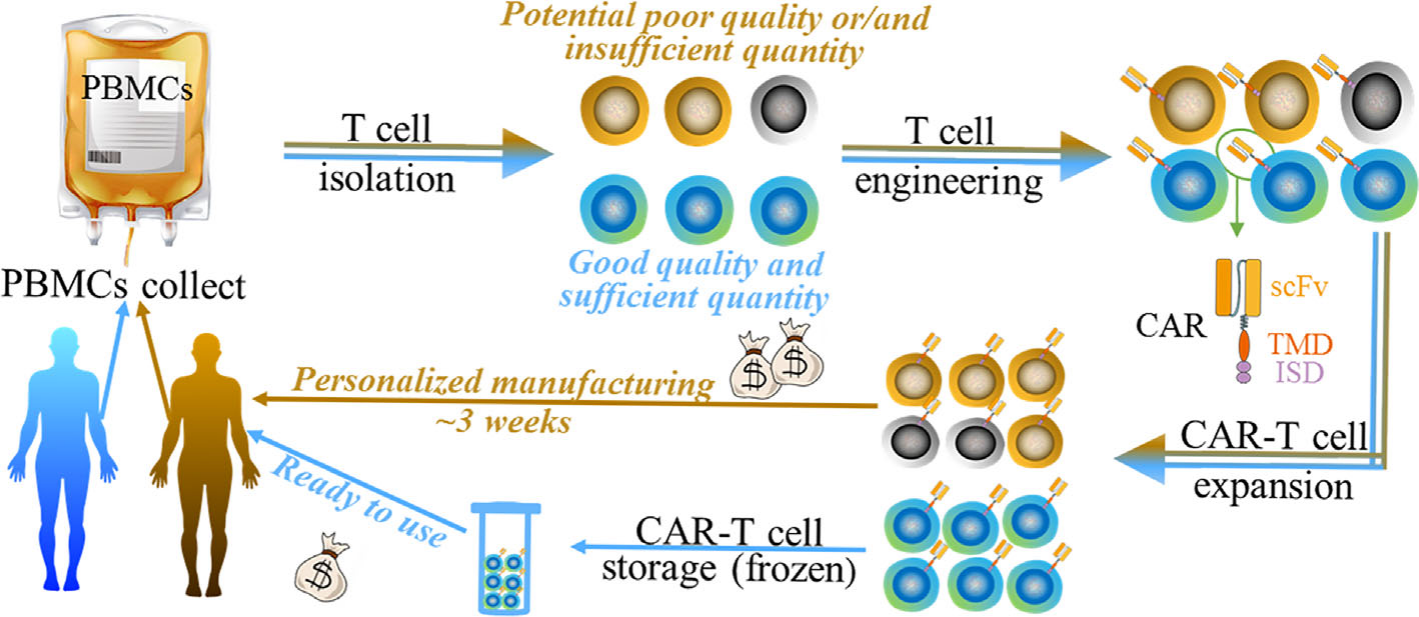
The generalized manufacturing procedure of autologous chimeric (brown arrow indicated) and UC therapy (blue arrow indicated). Both of them undergo 1) PBMC collect; 2) T cell isolation; 3) T cell engineering; 4) CAR-T cell expansion, and 5) CAR-T cell storage (only for UC therapy). Autologous CAR product may have disadvantages such as money-, time- consuming, and the potential poor quality/quantity of patient-derived cells (black/grey cells indicated). UC therapy product has various potential benefits such as simplifying and standardizing the manufacturing at relatively lower cost.

CAR represents a type of engineered receptors that is composed of a single-chain variable fragment (scFv) targeting tumor-associated-antigens (TAAs) or specific antigens for certain types of cells (e.g. CD19 on B cells), a transmembrane domain (TMD), and an intracellular signaling domain (ISD) (9) (**Figure 1**). It bestows the recipient immune (commonly T) cells with enhanced anti-tumor activity, leading to the profound elimination of tumor cells and preventing the tumor relapse by promoting immune surveillance. Currently, T cells used in CAR therapy are mainly isolated from the peripheral blood mononuclear cells (PBMCs) of patients. CAR is subsequently grafted onto the T cells, and the CAR-T cells are expanded in sophisticated culture conditions *in vitro*. Lastly, these autologous CAR-T products are re-infused into the bloodstream of the patient *in vivo* (10) (**Figure 1**).

Although CAR therapy has made clinical progress recently, the use is limited by multiple issues such as severe toxicity (e.g. cytokine release syndrome, CRS) (11, 12), safety (on-target, off-tumor response) (13, 14), and disease relapse (15, 16). These issues have been summarized in various reviews. We hereby focus on another important issue—the universality of CAR therapy. Although the autologous CAR-T cell therapy has gained outstanding clinical results with advantages such as the absence of allogeneic reaction and long persistence (17), its intrinsic disadvantages severely limit its broader applicability (**Figure 1**). Firstly, the personalized therapeutic approach remains at an unaffordable price [e.g. $475,000 for Kymriah **(** 7 **)**] for normal family and lays a heavy financial burden on a health care system. Secondly, the long manufacturing process, approximately 3 weeks (18), can be problematic for patients who suffer highly proliferative diseases such as acute leukemia. Patients may experience a rapid disease progression before the autologous CAR-T cell manufacturing is completed. Thirdly, as disease progression or other anti-tumor therapies such as chemotherapy or radiotherapy, the quality and quantity of patient-derived T cells may not meet the requirements. Furthermore, some individuals, such as infants or lymphogenic patients, cannot provide a sufficient quantity of immune cells for the manufacturing process (19). Therefore, the efficacy of such personalized CAR-T therapy may vary with individuals, making it difficult to be accurately evaluated.

The limitations of autologous CAR therapy thus bring the rise of a universal CAR strategy in this field. Therapeutic cells from healthy donors lay a foundation of good, controllable quality and sufficient quantity of the starting materials, making the scaleup industrialization process possible. More importantly, the ready-to-use cell products offer an immediately available treatment for patients at a significantly lower cost. The simplified and standardized manufacturing process gives the opportunity of using batches of products, which makes CAR treatment accord with a stable standard.

To construct a universal CAR (UC) system based on cells from healthy individuals, intense efforts are required to solve the problems related to “universal” cells, i.e. allogeneic cells, transplantation. Generally, two major issues should be carefully taken into consideration: GVHD and HVGA. The former is a serious complication and may be life-threatening, as donor cells will attack and damage host cells. The latter leads to the short persistence of donor CAR cells as they may be rejected by host, which will consequently limit the CAR-specific anti-tumor efficacy. This review will highlight current progress in the UC therapy in regards to GVHD and HVGA, additionally demonstrate a universal CAR design that may be applied to allogeneic cells, and finally document current clinical trials.

## Universal Cell (Allogeneic Cell) Transplantation Related Issues

**Figure 2:**
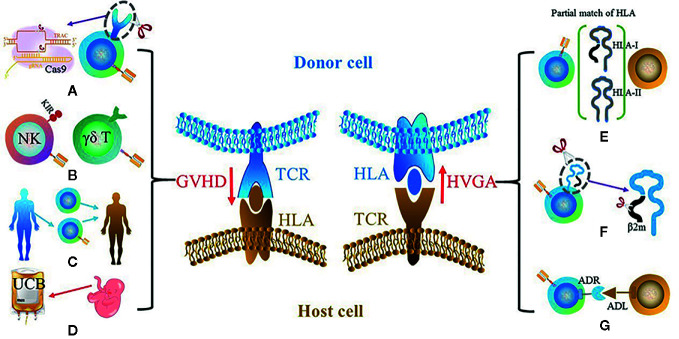
Two key barriers for UC therapy: GVHD (left) and HVGA (right). The main solutions for GVHD include **(A)** using gene-edited αβ T cells such as the knockout of αβ TCR using CRISPR-Cas9; **(B)** using other types of cells instead of αβ T cells such as NK cell and γδ T cell; **(C)** using donor-derived CAR cells; **(D)** using cells with different sources instead of PBMCs such as UCBs. The potential solutions for HVGA include **(E)** using HLA-matched donor, especially HLA-A and HLA-B that belong to HLA-I and HLA-DR that belongs to HLA-II; **(F)** using gene editing to knockout HLA of donor cells; **(G)** grafting an ADR that recognizes the ADL on host cells.

GVHD and HVGA mainly result from the disparity of human leukocyte antigens (HLAs) between donor and host cells. GVHD in allogeneic CAR cell transplantation can be severe and life-threatening, and serves as the primary cause of mortality. HVGA rejects allo-CAR cells, leading to reduced persistence and antitumor efficacy. Although partially attributed to the CAR design (20), the incidences of both GVHD and HVGA are mainly caused by allo-αβ T cells (21, 22). The T cell receptors (TCRs) in αβ T cells are responsible for recognizing peptides that are presented by HLAs. As the most polymorphic region in genome, the HLA locus bears thousands of expressed HLA variants. TCR repertoire in αβ T cells is constructed by the positive and negative selection processes during thymic selection, thus to be tolerant towards self-HLA. However, the donor HLA molecules absent in thymic selection in the host will be regarded as foreign antigens, and *vice versa* (**Figure 2**). Therefore, the T cells from host and donor will attack each other, which leads to GVHD or/and HVGA. The following section will document the current development to reduce or eliminate GVHD and HVGA, touching on the further optimization in allogeneic UC therapy.

### How to Reduce/Eliminate GVHD

#### Gene-Edited αβ-T Cells

Owing to the advanced gene editing technology, αβ TCR can be eliminated and αβ TCR^−^ T cells (abbreviated as TCR^−^ T below in this section) can be used in CAR therapy without GVHD (**Figure 2**). The common basis of this strategy is to disrupt the αβ TCR gene loci of donor T cells, which eliminates the HLA-dependent recognition and abolishes GVHD. The disruption of αβ TCR can be performed in either α or β subunit of TCR. However, as the α subunit contains only one constant region, the disruption of the gene loci that encodes constant α chain (TRAC) is the commonly used strategy for ablating αβ TCR complex and firstly reported in 2012 (23).

Several gene editing methods can be used to achieve this aim, such as zinc finger nuclease (ZFN) (24), transcription activator-like effector nuclease (TALEN) (25), and clustered regularly interspaced short palindromic repeats (CRISPR)-CRISPR associated protein 9 (Cas9) system (26). These gene editing methods share a common goal of producing a specific DNA double-strand break (DSB) at a specific site firstly. Followed by the generation of DSB, the intrinsic cellular DNA repair mechanism is triggered, leading to the gene inactivation (gene knockout) *via* either error-prone non-homologous end-joining pathway or homologous recombination pathway. In some cases, CAR can be precisely inserted (gene knock-in) in the DSB site such as using adeno-associated virus (AAV) *via* the homologous recombination pathway.

The discovery of ZFN in 1990 marked the era of gene editing. ZFN used a zinc finger motif to bind DNA triplets, and the FokI nuclease to cleave DNA (27, 28). However, as each zinc finger repeats recognizes only three bases, the off-target mutations and cleavages are very high. Therefore, TALEN and CRISPR systems have been more widely used in recent researches. TALEN is based on proteins that are derived from TALEs. TALEs are DNA-binding proteins with an array of >30 amino acid repeats. Each repeat is highly conserved, however, with the exception of two repeat variable di-residues (RVDs) at positions 12 and 13. The RVDs direct the FokI nuclease to cleave DNA and create DSB. As the length of the binding site is long (30+), TALEN shows much higher specificity (29, 30) and is the first gene-editing tool to produce TCR^−^ T cells. In 2015, Poirot et al. and Qasim et al. used TALEN to produce TCR^−^CD52^−^ CD19-CAR T cells (31, 32). The deficiency of αβ TCR abolished the GVHD reaction, and the knockout of CD52 rendered the CAR-T cell resistant to alemtuzumab (an anti-CD52 monoclonal antibody that is used to eliminate host T cells). This was the first time to prove that gene-edited allogeneic CAR-T cells can kill the tumor without introducing GVHD in NSG mouse model. Following this work, Qasim et al. used TCR^−^CD52^−^ CD19-CAR T cells to cure two infants with B cell acute lymphoblastic leukemia (B-ALL) (33). Both infants achieved a complete response with negative minimal residual disease, and without significant GVHD.

Apart from TALENs, CRISPR/Cas9 system is another widely used gene editing tool for producing desirable T cells. Unlike ZFN and TALEN systems that use protein domain to target DNA sequences, CRISPR/Cas9 uses RNA to recognize the desired gene locus. A guide-RNA (gRNA) that targets 20-nucleotide sequences guides Cas9 to bind the fixed protospacer adjacent motif (PAM). Cas9 subsequently cleavages the DNA at the targeted site (34, 35). Although the specificity of CRISPR/Cas9 is not as high as that of TALEN, the CRISPR/Cas9 system has several advantages such as easy-to-use and high efficiency. In 2017, Eyquem et al. used CRISPR-Cas9 to disrupt the TRAC locus, and AAV to carry the CAR construct flanked by DNA sequences homologous to both sides of the cutting site to insert CAR accurately into the desired locus (36). The one-step of knockout and knock-in allowed the high selectivity for TRAC integration and the absence of off-target hotspots. This integration resulted in averting clonal expansion and unifying the transgene expression. Further, the TRAC-CAR avoided tonic CAR signaling, internalized CAR expression, delayed the differentiation and exhaustion of effector T-cell, and enhanced the anti-tumor capability. Recently, the optimization of Cas9 based gene editing technology has further advanced the field of cell editing. Such improved technologies have been developed in several studies, including Cas9-HF1 (37) and CRISPR GUARD (38) system, which will be anticipated to apply in the future production of UC.

Although the gene editing CAR-T strategy has been validated in many preclinical and clinical trials, potential risks related to gene editing tools and biological characteristics of TCR^-^CAR-T cells remain poorly understood. Regarding gene editing tools, off-target cleavages may lead to DSBs at multiple locations that may cause unwanted translocations. Thus, unpredictable gene activation or/and inactivation or/and rearrangement may happen, which in turn leads to an unpredictable result of CAR-T cells. Another concern of this approach is the unknown biological characteristics of TCR^-^CAR-T cells. Firstly, few studies have reported the *in vivo* expansion of TCR^-^CAR-T cells, though the *in vitro* proliferation of TCR^-^CAR-T cells can be driven by the activation of CAR itself via contacting target antigens (36) and/or by some cytokines such as IL-7 and IL-15 (39). Secondly, whether the deficiency of TCR impacts the efficacy of TCR^-^CAR-T cells is controversial. Bridgeman et al. reported that the complex of CAR and endogenous TCR was beneficial for the T cell activation and the optimal effect of CAR-T cells (40). However, Yang et al. argued that concomitant activation of the CAR and TCR dramatically attenuated the efficacy of CD8^+^CAR-T cells *in vivo*, which led to the exhaustion and apoptosis of CD8^+^ CAR-T cell (41). These controversial reports suggested the possible association between TCR and the efficacy of CAR, however, no clear conclusion has been reached currently. This gap may be due to the unknown mechanism of how TCR impacts the biological characteristics of CAR-T cells, such as how TCR impacts tonic signaling and how such signaling affects the efficacy of CAR cells (42–45). Therefore, a deeper understanding of the biological characteristics of TCR^-^CAR-T cells and the underlying mechanism, especially *in vivo*, is required.

#### Other Types of Cells Instead of αβ-T Cell

The use of other types of immune cells, instead of αβ T cells, can be another possible approach to avoid GVHD (**Figure 2**). Natural killer (NK) cells are innate immune cells with an anti-tumor effect. Once sensing a proximal cell with an oncogenic marker, NK cells can be activated and eliminate tumor cells by direct cytotoxic activity or secreting numerous cytokines, chemokines, and growth factors. Unfortunately, NK cells frequently remain in a dysfunctional state in tumors, which indicates that tumor cells have evolved to escape NK-mediated killing. Although NK cells have been proved to provide a graft-*versus*-tumor (GVT) effect in a mouse model with ALL (46). A simple infusion of NK cells has shown dissatisfactory result in human: Rosenberg’s group injected unmodified autologous NK cells into eight patients with renal cell carcinoma or metastatic melanoma, but without any clinical responses (47); Burns et al. tried to treat the relapsed lymphoma or metastatic breast cancer using an NK-enriched leukapheresis product, unfortunately, showing to be ineffective (48). Recently, researches have shown that NK cells that are equipped with CAR can reinforce their tumor-killing activities and overcome HLA-mediated inhibitory signals, thus sparked considerable interest in using CAR-NK. In 2015, Campana’s group constructed a CD19-CAR-NK cell using CD8α as TMD coupled with CD3ζ and 4-1BB as ICD, which highly improved NK-mediated leukemic cell death (49). In the same year, Yu’s laboratory transduced human NK-92 cell line with a lentiviral construct that harbored a CAR targeting epidermal growth factor receptor (EGFR), which showed to be effective in the inhibition of glioblastoma growth and prolonged tumor-bearing mice survival (50). Nowadays, more studies have shown that the effective efficacies of CAR-NKs in targeting CD20 (51, 52), CD138 (53), CD3 (54), and CD5 (55). However, there are still some challenges that remain. One of them is to define the “memory” feature of CAR-NK cells *in vivo*. It has been evident that the transfer of naïve and memory cells showed a better outcome than the transfer of effector cells in the ACT (56). Increasing evidence has shown that NK cells have trained immunity (57) that differ from classical immunological memory, and can be induced by IL-12 (58), IL-15 (59), and IL-18 (60). The induced trained immunity feature renders NK cells with enhanced cytotoxic activity such as in the defense of CMV infection (61). Recent clinical trials showed that CAR-NK cells with trained immunity exhibited an enhanced anti-tumor response and induced complete remissions in patients with leukemia (62). Additionally, killer cell immunoglobulin-like receptors (KIRs) may be another reason that limits the universality of CAR-NK. KIRs are expressed in NK cells and play pivotal roles in the licensing and activation of NK cells by interacting with self HLA (63). The great diversity of active and/or inhibitory KIRs and HLAs leads to tremendous NK cell diversity. More importantly, tumor cells expressing KIRs ligands may inhibit the functions of some NK cell subsets *via* inhibitory KIRs. Additionally, it was observed that there was a broad inter-individual disparity in NK cell response against the same target as reported by Makanga et al (64). Therefore, in order to guide the selection of suitable NK subtypes from different donors, it is necessary to have a deeper understanding of the relationship between NK receptors and tumors. Alternatively, some studies pointed that the use of a cell line such as NK92 (65) may circumvent this problem, which has been successfully applied in some clinical trials and shown promising outcomes (discussed below). However, the intrinsic risk of NK cell lines, including potential *in vivo* tumorigenicity and the risk to induce the allo-immune responses of T and B cells, cannot be neglected. Apart from these limitations that result from the biological characteristics of NK cells, some challenges of CAR-NK relate to the manufacturing process such as low transduction efficacy, the very likely contamination of T or B cells in NK cell products, and low retained activity after recovery from a frozen state. In response, optimizations have been developed, such as a novel transduction method [e.g. electroporation (66)], using NK cells with different resource [e.g. cord blood (67)] and improving culturing condition [e.g. the addition of IL-2 (68) and IL-15 (59)].

In addition to NK cells, another possible candidate is γδ T cells that are capable of killing an array of cancerous cells including leukemia, lymphoma, and solid tumor cells (**Figure 2**) (69). γδ T cells are unlikely to induce GVHD as their TCRs are activated in an HLA-independent manner (70). Although γδ T cells account for a relatively smaller population (1~10%, albeit varying with individuals and ages) of T lymphocytes in PBMCs, they are more enriched at barrier sites such as ~20% in the colon. Of note, γδ T cells were observed as the most favorable prognostic parameter among 22 lymphocyte subsets (71). The predominant population in PBMCs, Vγ9Vδ2-TCR, has consistently shown to indicate a better outcome in various cancers such as prostate carcinoma, colorectal carcinoma and acute myeloblastic leukemia (72). Like NK cells, grafting CAR onto γδ T cells can enhance their cytotoxicity. The first CAR-γδ T design was firstly reported in 2004, and demonstrated the enhancement of antigen-specific tumor reactivity of GD2/CD19-CAR-γδ T (73). So far, Adicet Bio, Inc and Cotomed Therapeutics are pursuing the CAR γδ T strategy and marked it as an “off-the-shelf CAR product.” However, there exist some barriers to the application of CAR-γδ T. Firstly, due to the low frequency and number of γδ T cells in PBMCs, it is time-consuming to obtain a sufficient quantity of cells *in vitro*. A serum-free protocol supplemented with zoledronic acid and IL-2 allowed an average-fold expansion of autologous γδ T in the range of 25~310 in 2 weeks (74). However, the long expansion time might lead to T cell exhaustion, which was observed in CAR-αβ T cells (75). Another issue of γδ CAR-T is its potential on-target, off-tumor response. In the common culture condition, using phosphoantigen (e.g. BrHpp) as stimulator (76), γδ T cells have a propensity to expand Vγ9Vδ2 population that can specifically recognize butyrophilin-3A (BTN3A) (77). Although BTN3A is expressed at a high level in multiple tumors such as acute myeloid leukemia (78), colon (79), and ovarian (80), it is also expressed in normal cells (81). Therefore, the preferable expansion of Vγ9Vδ2 poses a potential on-target, off-tumor risk that enables γδ T cells to attack normal cells.

#### Donor-Derived CAR Cells

Instead of using CAR cells from irrelative donors, one approach is to use CAR cells that are derived from a stem cell/bone marrow cell transplant donor (**Figure 2**). A recent clinical report (82) showed that 8/20 patients with B cell malignancy achieved complete (6/8) or partial remission (2/8) after infusion CD19-CAR-T cells, along with the unmanipulated lymphocytes from the same donor. No new-onset acute GVHD (some patients had developed GVHD in previous lines of treatment), but only mild chronic ocular GVHD (5/20) occurred in the treatment. The reasons for reduced/none GVHD might be the shorter persistence of CAR-T cells [fewer than a median 4 weeks (83, 84)] and limited cell doses [1.2–3 fold less than the threshold cell dose (85, 86)]. This data was in line with previous clinical trials registered as NCT00840853 (87) and NCT01087294 (88), which validated the efficacy of donor-derived CAR-T cell therapy with reduced/non GVHD. However, there still existed contradictory reports that would raise the concern of the incidence of GVHD using this approach. For example, a patient with relapsed B-ALL developed an acute gastrointestinal GVHD grade 3 after the infusion of donor-derived CD19-CAR T cells (89). A study of refractory B-ALL reported half (6/11) patients developed skin GVHD grade 2 and one patient developed liver GVHD grade 2, after receiving allogeneic donor-derived CD-19 CAR T treatment (90). Thus, understanding the underlying mechanism about the relationship between donor-derived CAR T cells and GVHD is required, which can explain the incurrence of GVHD and provide a guideline for preventing GVHD in this approach. In addition to mechanism study, the optimization of donor-derived CAR cells may reduce GVHD. Wiebking et al. reported that NSG mice with ALL were cured after receiving the allogeneic hematopoietic stem cell and CD19-TCR^-^CAR-T from the same donor (91). The knockout of TCR by CRISPR-Cas9 in donor-derived CAR-T cells completely avoided the incidence of GVHD.

#### Cells With Different Sources Instead of PBMCs

One method of reducing or avoiding GVHD is to use cells with specific HLA expression pattern, instead of using cells derived from PBMCs (**Figure 2**). Such cell sources may include umbilical cord blood (CB), placenta-derived stem cells, and induced pluripotent stem cells (iPSCs). CB may provide an alternative resource of CAR cells with reduced GVHD, taking advantage of low immunogenicity (92). Both acute and chronic GVHD at ranges of grades were significantly lower after transplanting CB (4-6/6 HLA matched) when compared to transplanting PBMCs (8/8 or 7/8 HLA matched) (93). The features of CB-T cells, such as antigen-naïve (94) and impaired nuclear factor of activated T cell (NFAT) signaling (95), may explain the low score of GVHD. More importantly, antigen-naive CB-T cells can be differentiated into memory effector T cells in the tumor environment, so as to show potent anti-tumor effect such as in ALL (96). In 2006, Cooper laboratory showed that CD19 CB-CAR-T mediated the regression in murine models (97). After a decade, June et al. reported a case in a child that achieved complete remission without GVHD after the treatment of CD19 CB-CAR-T cells (98), albeit followed by a tumor relapse unfortunately. More recently, a few patents regarding the CB-CAR therapy have been approved, such as a CB-CAR-CD8^+^ T product for the prevention of acquired immunodeficiency syndrome (AIDS) associated lymphoma (Patent No. CN10960965-A). However, the limited number of nucleated cells in CB may hinder the broad application of CB-CAR therapy. This limitation may be solved by advanced technology that enables hundreds of expansions *in vitro* based on Good Manufacturing Practice (GMP) (99). For example, CB-CAR-T cells with central memory/effector phenotype can be expanded for more than 150-fold in the presence of IL-12 and IL-15, of note, showing enhanced anti-tumor efficacy *in vitro* and *in vivo* (100).

Compared to CBs, the other two cell resources (placenta-derived stem cells and iPSCs) mentioned above have been seldom reported, and without sufficient clinical assessment. Placenta-derived stem cells may be a potential cell resource for their HLA expression patterns, such as syncytiotrophoblast that displays HLA negative (101). The use of iPSCs has potential advantages such as their outstanding capabilities of self-renewal, possibilities of generating a bank of iPSCs with common HLA haplotypes and homogeneities. However, the differentiation of iPSCs into mature T cells has been challenging (102). So far, the use of these two types of cells lacks solid practical evidence. Only one patent claimed to use placental CAR-T cells for the treatment of cancers especially B cell cancer in 2020 (Patent No. WO2020113234-A1).

### How to Reduce/Eliminate HVGA Mediated Rejection

In addition to GVHD, HVGA is another important barrier for allogeneic UC therapy. As mentioned above, the disparity of HLA is the most significant reason for the host-mediated rejection of allogeneic cells. Apart from αβ T cells, NK cells or γδ T cells can still be eliminated by host immune cells due to the expression of foreign HLA on the cell surface. Therefore, the strategies aiming to avoid HVGA are necessary for almost all allogeneic CAR cells.

Allogeneic cell transplantation studies have shown that the most important HLA alleles to match for are HLA-A and HLA-B belonging to HLA class I, and HLA-DR belonging to HLA class II (103, 104). Matching for these loci can significantly reduce the occurrence of HVGA-mediated rejection. Therefore, selecting donors with matched HLA-A, HLA-B, and HLA-DR is a direct approach to avoid the rejection of allogeneic CAR cells (**Figure 2**). Taylor et al. have provided a theoretical calculation to show that a tissue bank from 150 homozygous HLA-typed donors could match above 90% of the UK population (105).

Instead of carefully selecting an HLA-matched donor, another strategy aims to make allogeneic cells “invisible” to the host immune system by disrupting HLA (**Figure 2**). The knockout of HLA can be achieved by disrupting related molecules that are necessary for the formation of functional HLA: β_2_-microglobulin (β2m) for HLA-I and transactivator (CIITA) or RFXANK for HLA-II (106). The concept has been validated in many studies such as reported by the Torikai group (107) and Choi group (108). However, the deficiency of HLA class I may lead donor cells to become more sensitive towards the recognition and destruction from recipient NK cells, as HLA molecules act as a type of ligand inhibitor of NK cells. Thus, the incidence of a host NK-mediated rejection may be another problem in HLA^−^ CAR cells. A solution for the NK-mediated rejection may be to insert HLA-I replacement molecules to bind the inhibitory receptors on NK cells. The HLA-I replacement molecules can be non-classical HLA molecules such as HLA-E or HLA-G (109), and siglect 7 or siglect 9 (110). However, these HLA replacements may be effective only for a subset of NK cells that express these receptors. In addition, few studies have validated whether the additional insertion will resist the NK-mediated rejection without reducing the efficacy of CAR cells.

Instead of matching or deleting HLA, Mo et al. engineered an allo-immune defense receptor (ADR) onto a CAR-T cell recently (111) (**Figure 2**). As activated lymphocytes (e.g. T cells and NK cells) would temporarily upregulate 4-1BB, a 4-1BB receptor was inserted in CAR-T cells to eliminate the activated alloreactive lymphocytes. Such design allowed the ADR-CAR-T cells resistant to the T- and NK cell–facilitated rejection *in vitro*. As a consequence, the ADR-CAR-T cells showed a longer persistence in mice with both hematopoietic and solid cancers. This design retained the normal expression of HLA and solved the potential risk of HLA-deficient donor cells, providing a new concept to solve the host rejection issue.

## Universal CAR Design

The current scFv on CAR cells is designed as a fixed antigen-recognition part. The rigid design narrows the therapeutic window of CAR as the incidence of TAAs escape/downregulation/loss in cancer treatment. Moreover, such design limits the application of CAR to a custom-made, as TAAs vary with individuals and diseases so that a complete manufacturing process is necessary for different patients. Therefore, coupled with allogeneic cells, a flexible and switchable design of CAR may contribute to the universality of CAR therapy.

To make a type of universal CAR cell that is suitable for more patients, the antigen-target portion of the scFv domain is removed, only leaving a fragment residue (FR) on the surface of CAR cells. Like conventional CAR cells, the FR connects the ICD *via* a TMD, but remains at a resting state as there is a lack of antigen-recognition process. The activation of FR-CAR will only be triggered after a stepwise addition of a free antibody that specifically recognizes both FR and TAAs. This design renders CAR cells with more flexibility and broader antigen specificities. Of note, the manufacturing of FR-CAR can be completed in advance and ready to use for different types of patients, if coupled with the modified allogeneic cells as discussed above.

Such universal CAR designs were initially reported in 2012 by Young et al (112) and Davila et al (113) groups, which were based on the mechanisms of avidin Vs biotin-antibody and fluorescein isothiocyanate (FITC) Vs anti-FITC-antibody respectively. Instead of a conventional scFv with a fixed antigen, Young used dimeric avidin (dcAv) without linking to any antibody as the FR on the surface of the T cell and connected to the ICD. Biotinylated antibody even at extremely low concentrations could successfully bind to dcAv with a high affinity (Kd = 10^−7^), permitting further immune-recognition towards TAAs. This in turn triggered the activation of CAR cells and mediated their anti-tumor activities. The efficacy of the FR-CAR was confirmed in an ovarian cancer xenograft mouse model. Similar research also supported the efficacy of the FR-CAR using an affinity-enhanced monomeric streptavidin 2 biotin-avidin system (114) in lustrum. Following the similar concept, Davila et al. tagged FR part with FITC, so that free tumor-targeting anti-FITC-antibody can bridge the FR-CAR and tumor. Moreover, recent researches have been making continuous efforts to design the FR-CAR system with different tags such as Fcγ (115) and peptide neo-epitopes (116), collectively and consistently support the reliability and efficacy of the universal FR-CAR. In 2018, Wong et al. proposed an upgraded version FR-CAR called “a split, universal, and programmable (SUPRA) CAR system” (117). The FR part of SUPRA-CAR consisted of a leucine zipper adaptor (zipCAR), which specifically bound to a tumor-targeting leucine zipper adaptor (zipFv). In addition to the ability to switch TAAs without a re-engineering process, the SUPRA-CAR could be “down-activated” or “turn-off” by adding non-tumor-targeting competitive leucine zippers that with varying affinities with zipCAR. Besides, the introduction of multi antigens with “OR” logic can be achieved by the addition of multi zipFvs. In summary, this design renders CAR products with more flexibility, as well as controllability during the treatment. Based on these breakthroughs in universal CAR design, future researches can further optimize the efficacy of such CAR with approaches such as engineering the tumor-targeting molecules [e.g. glycosylation (118) and PEGylation (119)] to prolong the serum half-life of FR-CAR cells.

Although the design of FR-CAR has several advantages, it should be noted that such design may bring some concerns that have not been investigated. The first issue is about the efficacy of FR-CAR. For example, the FR-CAR with avidin tag may recognize the membrane-bound biotinylated antibodies, as biotin is naturally present in humans with a plasma concentration ranging from 0.2 to 2 nM (120). This may block FR-CAR binding to added biotinylated antibodies and reduce its anti-tumor efficacy. In addition, as the expansion of the FR-CAR cells may need the continuous stimulation of antigens especially if using allogeneic TCR^-^ CAR-T cells, the repeated and high dosage of free antibody are necessary during the treatment. The pharmacokinetics of the antibody might be associated with the efficacy and safety of the FR-CAR product (121), which needs further exploration. The second issue is about the safety of FR-CAR. Some tags may not be naturally present in humans, however, the introduction of them may lead to new antigenicity. For example, FITC can lead to an early inflammatory response and develop to a fibrotic response that can maintain up to six months (122). Therefore, without careful consideration of the safety of these tags, the FR-CAR may have serious side effects. Finally, there are no clinical trials that have been conducted for such CAR product so far. To launch clinical trials as soon as possible, more preclinical data about such CAR therapy was urgently necessary.

## Clinical Trials of UC Therapy

The momentum of CAR therapy has been generated when the FDA approved the first CAR-T product. The translation of CAR therapy is global, with more than 500 clinical trials listed on ClinicalTrial.gov. As available clinical cell therapies continue to rise and gene-editing technology offers ground-breaking opportunities, the clinical trials of universal CAR started after the year 2016. As of October 2020, there are 36 clinical trials (**Table 1**, excluding the withdrawn ones) that have been registered in ClinicalTrial.gov, with most of them aim at treating hematological, lymphomas, and myeloma malignancies. Recently, a clinical trial regarding the coronavirus disease 2019 (COVID-19) was raised (NCT04324996), targeting the S protein of SARS-CoV-2 and NKG2DL on the surface of infected cells. This case strongly indicates that a ready-to-use allogeneic cell product may be the practical option in such a pandemic situation, rather than a patient-derived cell product.

**Table 1:** Current clinical trials of UC therapy.

NCT Number****	Conditions****	****Target	Cell type edited****	Phases****	Start Date****
NCT03366350	ALL, B-CLP	CD19	T	I/II	Apr-16
NCT02808442	Pediatric RR B-ALL	CD19	T	I	Jun-16
NCT02746952	B-ALL	CD19	T	I	Aug-16
NCT02735083	Advanced lymphoid malignancies	CD19	T	I	Sep-16
NCT03114670	Adult AML	CD123	T	I	Mar-17
NCT03166878	B-CLK, B-CLP	CD19	T	I/II	Jun-17
NCT03203369	AML	CD123	T	I	Jun-17
NCT03190278	RR AML	CD123	T	I	Jun-17
NCT03463928	B-CLK	CD19-22/CD19	Donor-derived T	I	Oct-17
NCT03398967	B-CLK, B-CLP	CD-19/CD20/CD22	T	I/II	Jan-18
NCT03545815	Mesothelin positive solid tumors	Mesothelin	T	I	Mar-18
NCT03692429	Colorectal cancer	NKG2D based CYAD-101	T	I	Nov-18
NCT03939026	RR B-CL, FL	CD19	T	I	May-19
NCT04035434	RR B-cell malignance	CD19	T	I	Jul-19
NCT04093596	RR myeloma	BCMA	T	I	Sep-19
NCT04150497	RR B-ALL	CD22	T	I	Oct-19
NCT04049513	LNHB, diffuse LB-CLP, PMBCL, TFL	CD19	T	I	Oct-19
NCT04142619	RR myeloma	CS1	T	I	Nov-19
NCT04230265	AML, B-ALL, BPDCN	CD123	T	I	Jan-20
NCT04264039	B-ALL, B-CLP	CD-19	T	Early I	Apr-20
NCT04264078	T cell leukemia, T-CL	CD-7	T	Early I	Apr-20
NCT04516551	RR adult ALL, B-CLK	CD19	T	I	Aug-20
NCT00995137	B-ALL	CD19	NK	I	Oct-09
NCT01974479	B-ALL	CD19	NK	I	Sep-13
NCT02742727	CD7 Positive leukemia and lymphoma	CD7	NK92	I/II	Mar-16
NCT02839954	MUC1 positive RR solid tumor	Muc1	NK92	I/II	Jul-16
NCT02892695	Leukemia and lymphoma	CD19	NK92	I/II	Sep-16
NCT02944162	RR AML	CD33	NK92	I/II	Oct-16
NCT03056339	B-CLP	CD19	CB-NK	I/II	Jun-17
NCT03383978	Glioblastoma	HER2	NK92	I	Dec-17
NCT03415100	Metastatic solid tumors	NKG2D	NK	I	Jan-18
NCT03656705	Non-small cell lung carcinoma	CCCR	NK92	I	Sep-18
NCT03940833	RR multiple myeloma	BMCA	NK92	I/II	May-19
NCT04004637	R/R NK/T-LP, T-LL, and ALL	CD7	NK92	I	Jun-19
NCT03579927	B-CLP	CD19	CB-NK	I/II	Oct-19
NCT04324996	COVID-19	NKG2D/ACE/NKG2D-ACE	NK	I/II	Mar-20
NCT04107142	RR solid tumors	NKG2D	γδ T	I	Dec-19

First clinical trials for allogeneic CAR-T therapy (e.g. NCT02808442 and NCT02746952) used TALEN to disrupt the TRAC locus of αβ T cells, with stringently capped carriage of residual αβ TCR-T cells to 5 × 10^4^ cells/kg. Based on the gene-edited approach and preclinical data reported by Qasim (33), two further clinical trials have shown promising results currently: 5/5 children achieved CR by days 28–42 after the therapeutical cell infusion (123); and 4/6 adults achieved CR by days 28 after the UCAR-T cells infusion at an escalated cell dose (124). In 2017, Cellectis’ second series of TALEN-edited cell therapy that depleted TRAC and targeted at CD123 was registered (NCT03190278), with the first patient dosed in 2020. This CAR product had an optimized CAR design: an scFv that specifically recognized CD123 and linked to 4-1BB and CD3ζ domain was constructed, with the RQR8 epitope marker/suicide gene incorporated (125, 126). This trial was a replacement of the Cellectis’ first UCART123 clinical trial, as the previous version was put on hold by FDA following the severe CRS in the first patient dosed (127). Concerning the safety issue about the on-target, off-tumor, the universal CAR-T may take advantage of its short persistence as CD123 is expressed on some normal HSCs, progenitors, and endothelial cells from small-caliber blood vessels (128, 129).

Apart from the TALEN system, CRISPR technology also plays an important role in producing allogeneic CAR-T cells. However, as CRISPR technology was discovered ~2 years after TALEN, fewer clinical trials have been registered so far. In 2019, an allogeneic CD19 CAR-T product was raised based on the CRISPR system (NCT04035434), with the depletion of TRAC and β2m. Of note, the advanced gene-editing technology makes the desirable cells account for 54–66% across five donors, indicating the promise for the future pipeline (130).

In addition to T cells, NK cells have been one of the most used candidates for UC therapy. As the intrinsic biological characteristics of NK cells are unlikely to induce GVHD, the allogeneic CAR-NK clinical trial started 7 years before the emergence of the allogeneic CAR-T clinical trials, and was firstly conducted at St. Jude Children’s Research Hospital in 2009 (NCT00995137) to treat CD19 B-ALL. Recently, scientists have focused on testing whether CAR-NK modality can keep up with its CAR-T counterparts in the perspectives of safety and efficacy. As one of the most significant safety concerns, CRS is mainly attributed to cytokine storm that is initiated by some pro-inflammatory cytokines such as tumor necrosis factor α, IL-1, and IL-6 (131). These cytokines are frequently released in the interaction of CAR-T cells and cancerous cells. On the contrary, the NK-mediated tumor elimination process mainly produces cytokines such as interferon γ (132) and granulocyte macrophage colony-stimulating factor (133) that will not cause severe CRS. In clinical trial NCT02944162, 5 × 10^9^ CD33 CAR-NK92 was injected into ten patients with RR-AML. Only three of them suffered mild fever and CRS after the infusion, and these symptoms disappeared the next day (134). In addition, the limited lifespan of NK cells in circulation adds to the safety profile of CAR-NK therapy. Compared to that allogeneic CAR-T cells can persist months, most allogeneic primary CAR-NK cells are rejected in 14 to 21 days after infusion, and CAR-NK92 cells do not expand and persist *in vivo* as they are irradiated in prior to be transferred. The safety profile of CAR-NK therapy has been validated in many studies, as exemplified that a high dose of NK92 (1 × 10^10^ cells/m^2^ body surface) can be tolerated in the clinic (65). It is noteworthy that a short persistence may not be beneficial for long-term remission of tumor cells, though it circumvents some safety issues. Redosing may be a solution to eradicate cancerous cells for short persistent allogeneic CAR-NK or CAR-T cells, however, such strategy must account for the risk of alloimmunization unless the same CAR cell batch. In this setting, NK92 may be advantageous as its high stability. Apart from the safety issue, another important question to be answered is the efficacy of CAR-NK. Compared to that CAR-T only triggers the demise of cancerous cells in the TAA-restricted manner, CAR-NK additionally eliminates cancerous cells in the TAA-unrestricted manner *via* a panel of natural cytotoxicity receptors (e.g. NKp30, NKp44, NKp46, and NKG2D) (135) and antibody-dependent cell mediated cytotoxicity (136). Given these features, CAR-NK cells not only possess an enhanced anti-tumor efficacy against normal tumor cells, but also kill tumor variants that lose TAAs. Interestingly, T cells showed higher antitumor cytotoxicity when they were equipped with NK receptors: the introduction of NKp46 onto T cells upregulated surface activation markers (CD25 and CD69) and improved the antitumor effect both *in vitro* and *in vivo* (137); the graft of NKG2D onto CAR-T cells additionally targeted immunosuppressive myeloid cells and T_reg_ cells that express the ligand of NKG2D (138, 139). Although these preclinical data are encouraging, the number of clinical trials of CAR-NK falls behind that of CAR-T therapy if the clinical trials of autologous CAR therapy are included. Thus, more clinical data and solid evidence are required to validate the actual safety and efficacy of CAR-NK products, both in the aspects of allogeneic primary CAR-NK and CAR-NK92.

Although a myriad of clinical trials has been launched, more cases to ascertain the efficacy and safety of therapy, as well as long-term follow up monitor are required. One of the greatest barriers in stepping clinical trials is the paucity of preclinical models, which leads to the lack of preclinical research and data. Most preclinical studies typically transferred human cells in murine xenograft models (140, 141). Thus, the interpretations may have a bias due to the lack of an immunocompetent environment, and prevents the long-term monitoring about GVHD, HVGA, and the persistence of CAR cells. In addition, it could be found that most UC therapies are for hematologic and related diseases. However, solid tumors cause the most cancer-related deaths. Several factors may result in the poor efficacies of CAR cells on solid tumors, such as the lack of tumor-specific targets, the immuno-suppressive tumor microenvironment, and the difficulty in homing and accessing to the tumor site (142–145).

## Conclusions

A UC therapy based on healthy individual cells may provide multiple advantages over personalized CAR therapy based on patient-derived cells. However, intense efforts are required to solve GVHD and HVGA. In this review, we have discussed that there are several approaches to reduce/eliminate GVHD: using gene-edited αβ TCR^-^ T cells, other types of cells such as NK and γδ T cells, donor-derived CAR cells, and cells with different sources instead of PBMCs. In addition, the main approaches to solve HVGA include using donor cells with a partial match of HLA, gene-edited HLA^-^ T cells, and ADR-CAR-T cells. Coupled with a flexible design of scFv, the universality of CAR therapy may further revolutionize the ACT field.

## Author Contributions

YZ drafted the main body of this manuscript. PL modified the manuscript. HF and GW drew the figures. XZ takes primary responsibility for this paper as the corresponding author. All authors contributed to the article and approved the submitted version.

## Funding 

This work was supported by the Major Research Plan of the National Natural Science Foundation of China (grant number 91742102) and the National Science and Technology Major Project of China (2018ZX10302206).

## Conflict of Interest

The authors declare that the research was conducted in the absence of any commercial or financial relationships that could be construed as a potential conflict of interest.

## Abbreviations

AAV, adeno-associated virus; ACT, adoptive cell therapy; ADR, allo-immune defense receptor; ALL, acute lymphoblastic leukemia; AML, acute myeloid leukemia; ASD, autism spectrum disorder; B-ALL, B cell-ALL; B-CLK, B Cell leukemia; B-CLP, B-cell lymphoma; β2m, β2-microglobulin; BTN3A, butyrophilin-3A; BPDCN, blastic plasmacytoid dendritic cell neoplasm; CAR, chimeric antigen receptor; CB, cord blood; COVID-19, coronavirus disease 2019; CRISPR, clustered regularly interspaced short palindromic repeats; CRS, cytokine release syndrome; DSB, double-strand break; EGFR, epidermal growth factor receptor; FL, follicular lymphoma; FR, fragment residue; GMP, good manufacturing practice; GVHD, graft-*versus*-host disease; GVT, graft-*versus*-tumor; HLA, human leukocyte antigen; HVGA, host-*versus*-graft activities; iPSCs, induced pluripotent stem cells; ISD, intracellular signaling domain; KIR, killer cell immunoglobulin-like receptor; LBCL, large B cell lymphoma; LL, lymphoblastic lymphoma; LNHB, lymphomas Non-Hodgkin's B-Cell; mAb, monoclonal antibody; NK, natural killer; PBMC, peripheral blood mononuclear cell; PMBCL, primary mediastinal B-cell lymphoma; RR, relapsed/refractory; scFv, single-chain variable fragment; TAA, tumor-associated-antigen; TALEN, transcription activator-like effector nuclease; T-CL, T-cell Lymphoma; TCR, T cell receptor; TFL, transformed follicular lymphoma; TILs, tumor-infiltrating lymphocytes; TMD, trans-membrane domain; TRAC, T cell receptor constant α chain; UC, universal CAR; ZFN, zinc finger nuclease.

## References

[1] Rosenberg SA Packard BS Aebersold PM Solomon D Topalian SL Toy ST Use of tumor-infiltrating lymphocytes and interleukin-2 in the immunotherapy of patients with metastatic melanoma. A preliminary report. N Engl J Med (1988) 319(25):1676–80. 10.1056/NEJM198812223192527 3264384

[2] Kolb HJ Mittermuller J Clemm C Holler E Ledderose G Brehm G Donor leukocyte transfusions for treatment of recurrent chronic myelogenous leukemia in marrow transplant patients. Blood (1990) 76(12):2462–5. 10.1182/blood.V76.12.2462.bloodjournal76122462 2265242

[3] Robert C Long GV Brady B Dutriaux C Maio M Mortier L Nivolumab in previously untreated melanoma without BRAF mutation. N Engl J Med (2015) 372(4):320–30. 10.1056/NEJMoa1412082 25399552

[4] Robert C Ribas A Schachter J Arance A Grob JJ Mortier L Pembrolizumab versus ipilimumab in advanced melanoma (KEYNOTE-006): post-hoc 5-year results from an open-label, multicentre, randomised, controlled, phase 3 study. Lancet Oncol (2019) 20(9):1239–51. 10.1016/S1470-2045(19)30388-2 31345627

[5] Hodi FS O’Day SJ McDermott DF Weber RW Sosman JA Haanen JB Improved survival with ipilimumab in patients with metastatic melanoma. N Engl J Med (2010) 363(8):711–23. 10.1056/NEJMoa1003466 PMC3549297 20525992

[6] Kantoff PW Higano CS Shore ND Berger ER Small EJ Penson DF Sipuleucel-T immunotherapy for castration-resistant prostate cancer. N Engl J Med (2010) 363(5):411–22. 10.1056/NEJMoa1001294 20818862

[7] Ali S Kjeken R Niederlaender C Markey G Saunders TS Opsata M The European Medicines Agency Review of Kymriah (Tisagenlecleucel) for the Treatment of Acute Lymphoblastic Leukemia and Diffuse Large B-Cell Lymphoma. Oncologist (2020) 25(20)e321–e7. 10.1634/theoncologist.2019-0233 PMC7011647 32043764

[8] Papadouli I Mueller-Berghaus J Beuneu C Ali S Hofner B Petavy F EMA Review of Axicabtagene Ciloleucel (Yescarta) for the Treatment of Diffuse Large B-Cell Lymphoma. Oncologist (2020) 25(10):894–902. 10.1634/theoncologist.2019-0646 PMC7543293 32339368

[9] Jensen MC Riddell SR Designing chimeric antigen receptors to effectively and safely target tumors. Curr Opin Immunol (2015) 33:9–15. 10.1016/j.coi.2015.01.002 25621840 PMC4397136

[10] June CH Sadelain M Chimeric Antigen Receptor Therapy. N Engl J Med (2018) 379(1):64–73. 10.1056/NEJMra1706169 29972754 PMC7433347

[11] Neelapu SS Tummala S Kebriaei P Wierda W Gutierrez C Locke FL Chimeric antigen receptor T-cell therapy - assessment and management of toxicities. Nat Rev Clin Oncol (2018) 15(1):47–62. 10.1038/nrclinonc.2017.148 28925994 PMC6733403

[12] Brudno JN Kochenderfer JN Toxicities of chimeric antigen receptor T cells: recognition and management. Blood (2016) 127(26):3321–30. 10.1182/blood-2016-04-703751 PMC4929924 27207799

[13] Yu S Yi M Qin S Wu K Next generation chimeric antigen receptor T cells: safety strategies to overcome toxicity. Mol Cancer (2019) 18(1):125. 10.1186/s12943-019-1057-4 31429760 PMC6701025

[14] D’Ippolito E Schober K Nauerth M Busch DH T cell engineering for adoptive T cell therapy: safety and receptor avidity. Cancer Immunol Immunother (2019) 68(10):1701–12. 10.1007/s00262-019-02395-9 PMC11028346 31542797

[15] Jain T Bar M Kansagra AJ Chong EA Hashmi SK Neelapu SS Use of Chimeric Antigen Receptor T Cell Therapy in Clinical Practice for Relapsed/Refractory Aggressive B Cell Non-Hodgkin Lymphoma: An Expert Panel Opinion from the American Society for Transplantation and Cellular Therapy. Biol Blood Marrow Transplant (2019) 25(12):2305–21. 10.1016/j.bbmt.2019.08.015 31446199

[16] Li X Chen W Mechanisms of failure of chimeric antigen receptor T-cell therapy. Curr Opin Hematol (2019) 26(6):427–33. 10.1097/MOH.0000000000000548 PMC6791516 31577606

[17] Ritchie DS Neeson PJ Khot A Peinert S Tai T Tainton K Persistence and efficacy of second generation CAR T cell against the LeY antigen in acute myeloid leukemia. Mol Ther (2013) 21(11):2122–9. 10.1038/mt.2013.154 PMC3831035 23831595

[18] Kohl U Arsenieva S Holzinger A Abken H CAR T Cells in Trials: Recent Achievements and Challenges that Remain in the Production of Modified T Cells for Clinical Applications. Hum Gene Ther (2018) 29(5):559–68. 10.1089/hum.2017.254 29620951

[19] Poehlein CH Haley DP Walker EB Fox BA Depletion of tumor-induced Treg prior to reconstitution rescues enhanced priming of tumor-specific, therapeutic effector T cells in lymphopenic hosts. Eur J Immunol (2009) 39(11):3121–33. 10.1002/eji.200939453 PMC2850261 19839008

[20] Ghosh A Smith M James SE Davila ML Velardi E Argyropoulos KV Donor CD19 CAR T cells exert potent graft-versus-lymphoma activity with diminished graft-versus-host activity. Nat Med (2017) 23(2):242–9. 10.1038/nm.4258 PMC5528161 28067900

[21] Radestad E Wikell H Engstrom M Watz E Sundberg B Thunberg S Alpha/beta T-cell depleted grafts as an immunological booster to treat graft failure after hematopoietic stem cell transplantation with HLA-matched related and unrelated donors. J Immunol Res (2014) 2014:578741. 10.1155/2014/578741 25371909 PMC4211312

[22] Abdelhakim H Abdel-Azim H Saad A Role of alphabeta T Cell Depletion in Prevention of Graft versus Host Disease. Biomedicines (2017) 5(3):35–48. 10.3390/biomedicines5030035 PMC5618293 28672883

[23] Torikai H Reik A Liu PQ Zhou Y Zhang L Maiti S A foundation for universal T-cell based immunotherapy: T cells engineered to express a CD19-specific chimeric-antigen-receptor and eliminate expression of endogenous TCR. Blood (2012) 119(24):5697–705. 10.1182/blood-2012-01-405365 PMC3382929 22535661

[24] Gaj T Guo J Kato Y Sirk SJ Barbas CF3 Targeted gene knockout by direct delivery of zinc-finger nuclease proteins. Nat Methods (2012) 9(8):805–7. 10.1038/nmeth.2030 PMC3424280 22751204

[25] Sun N Zhao H Transcription activator-like effector nucleases (TALENs): a highly efficient and versatile tool for genome editing. Biotechnol Bioeng (2013) 110(7):1811–21. 10.1002/bit.24890 23508559

[26] Doench JG Hartenian E Graham DB Tothova Z Hegde M Smith I Rational design of highly active sgRNAs for CRISPR-Cas9-mediated gene inactivation. Nat Biotechnol (2014) 32(12):1262–7. 10.1038/nbt.3026 PMC4262738 25184501

[27] Kim YG Cha J Chandrasegaran S Hybrid restriction enzymes: zinc finger fusions to Fok I cleavage domain. Proc Natl Acad Sci U S A (1996) 93(3):1156–60. 10.1073/pnas.93.3.1156 PMC40048 8577732

[28] Smith J Bibikova M Whitby FG Reddy AR Chandrasegaran S Carroll D Requirements for double-strand cleavage by chimeric restriction enzymes with zinc finger DNA-recognition domains. Nucleic Acids Res (2000) 28(17):3361–9. 10.1093/nar/28.17.3361 PMC110700 10954606

[29] Boch J Scholze H Schornack S Landgraf A Hahn S Kay S Breaking the code of DNA binding specificity of TAL-type III effectors. Science (2009) 326(5959):1509–12. 10.1126/science.1178811 19933107

[30] Bogdanove AJ Voytas DF TAL effectors: customizable proteins for DNA targeting. Science (2011) 333(6051):1843–6. 10.1126/science.1204094 21960622

[31] Poirot L Philip B Schiffer-Mannioui C Le Clerre D Chion-Sotinel I Derniame S Multiplex Genome-Edited T-cell Manufacturing Platform for “Off-the-Shelf” Adoptive T-cell Immunotherapies. Cancer Res (2015) 75(18):3853–64. 10.1158/0008-5472.CAN-14-3321 26183927

[32] Qasim W Amrolia PJ Samarasinghe S Ghorashian S Zhan H Stafford S First Clinical Application of Talen Engineered Universal CAR19 T Cells in B-ALL. Blood (2015) 126(23):2046–. 10.1182/blood.V126.23.2046.2046

[33] Qasim W Zhan H Samarasinghe S Adams S Amrolia P Stafford S Molecular remission of infant B-ALL after infusion of universal TALEN gene-edited CAR T cells. Sci Transl Med (2017) 9(374):eaaj2013. 10.1126/scitranslmed.aaj2013 28123068

[34] Jinek M Chylinski K Fonfara I Hauer M Doudna JA Charpentier E A programmable dual-RNA-guided DNA endonuclease in adaptive bacterial immunity. Science (2012) 337(6096):816–21. 10.1126/science.1225829 PMC6286148 22745249

[35] Ran FA Hsu PD Wright J Agarwala V Scott DA Zhang F Genome engineering using the CRISPR-Cas9 system. Nat Protoc (2013) 8(11):2281–308. 10.1038/nprot.2013.143 PMC3969860 24157548

[36] Eyquem J Mansilla-Soto J Giavridis T van der Stegen SJ Hamieh M Cunanan KM Targeting a CAR to the TRAC locus with CRISPR/Cas9 enhances tumour rejection. Nature (2017) 543(7643):113–7. 10.1038/nature21405 PMC5558614 28225754

[37] Kleinstiver BP Pattanayak V Prew MS Tsai SQ Nguyen NT Zheng Z High-fidelity CRISPR-Cas9 nucleases with no detectable genome-wide off-target effects. Nature (2016) 529(7587):490–5. 10.1038/nature16526 PMC4851738 26735016

[38] Coelho MA De Braekeleer E Firth M Bista M Lukasiak S Taylor BJM CRISPR GUARD protects off-target sites from Cas9 nuclease activity using short guide RNAs. Nat Commun (2020) 11:4132–43. 10.1038/s41467-020-17952-5 PMC7431537 32807781

[39] Rubinstein MP Lind NA Purton JF Filippou P Best JA McGhee PA IL-7 and IL-15 differentially regulate CD8+ T-cell subsets during contraction of the immune response. Blood (2008) 112(9):3704–12. 10.1182/blood-2008-06-160945 PMC2572798 18689546

[40] Bridgeman JS Hawkins RE Bagley S Blaylock M Holland M Gilham DE The optimal antigen response of chimeric antigen receptors harboring the CD3zeta transmembrane domain is dependent upon incorporation of the receptor into the endogenous TCR/CD3 complex. J Immunol (2010) 184(12):6938–49. 10.4049/jimmunol.0901766 20483753

[41] Yang Y Kohler ME Chien CD Sauter CT Jacoby E Yan C TCR engagement negatively affects CD8 but not CD4 CAR T cell expansion and leukemic clearance. Sci Transl Med (2017) 9(417):eaag1209. 10.1126/scitranslmed.aag1209 29167392 PMC6944272

[42] Milone MC Fish JD Carpenito C Carroll RG Binder GK Teachey D Chimeric receptors containing CD137 signal transduction domains mediate enhanced survival of T cells and increased antileukemic efficacy in vivo. Mol Ther (2009) 17(8):1453–64. 10.1038/mt.2009.83 PMC2805264 19384291

[43] Qin L Lai Y Zhao R Wei X Weng J Lai P Incorporation of a hinge domain improves the expansion of chimeric antigen receptor T cells. J Hematol Oncol (2017) 10(1):68. 10.1186/s13045-017-0437-8 28288656 PMC5347831

[44] Gomes-Silva D Mukherjee M Srinivasan M Krenciute G Dakhova O Zheng Y Tonic 4-1BB Costimulation in Chimeric Antigen Receptors Impedes T Cell Survival and Is Vector-Dependent. Cell Rep (2017) 21(1):17–26. 10.1016/j.celrep.2017.09.015 28978471 PMC5645034

[45] Diogo Gomes da Silva MM Srinivasan M Dakhova O Liu H Grilley B Gee AP Direct Comparison of In Vivo Fate of Second and Third-Generation CD19-Specific Chimeric Antigen Receptor (CAR)-T Cells in Patients with B-Cell Lymphoma: Reversal of Toxicity from Tonic Signaling Blood. Blood (2016) 22(128):1851–1851. 10.1182/blood.V128.22.1851.1851

[46] Olson JA Leveson-Gower DB Gill S Baker J Beilhack A Negrin RS NK cells mediate reduction of GVHD by inhibiting activated, alloreactive T cells while retaining GVT effects. Blood (2010) 115(21):4293–301. 10.1182/blood-2009-05-222190 PMC2879101 20233969

[47] Parkhurst MR Riley JP Dudley ME Rosenberg SA Adoptive transfer of autologous natural killer cells leads to high levels of circulating natural killer cells but does not mediate tumor regression. Clin Cancer Res (2011) 17(19):6287–97. 10.1158/1078-0432.CCR-11-1347 PMC3186830 21844012

[48] Burns LJ Weisdorf DJ DeFor TE Vesole DH Repka TL Blazar BR IL-2-based immunotherapy after autologous transplantation for lymphoma and breast cancer induces immune activation and cytokine release: a phase I/II trial. Bone Marrow Transplant (2003) 32(2):177–86. 10.1038/sj.bmt.1704086 12838283

[49] Imai C Iwamoto S Campana D Genetic modification of primary natural killer cells overcomes inhibitory signals and induces specific killing of leukemic cells. Blood (2005) 106(1):376–83. 10.1182/blood-2004-12-4797 PMC1895123 15755898

[50] Han J Chu J Keung Chan W Zhang J Wang Y Cohen JB CAR-Engineered NK Cells Targeting Wild-Type EGFR and EGFRvIII Enhance Killing of Glioblastoma and Patient-Derived Glioblastoma Stem Cells. Sci Rep (2015) 5:11483. 10.1038/srep11483 26155832 PMC4496728

[51] Chu Y Hochberg J Yahr A Ayello J van de Ven C Barth M Targeting CD20+ Aggressive B-cell Non-Hodgkin Lymphoma by Anti-CD20 CAR mRNA-Modified Expanded Natural Killer Cells In Vitro and in NSG Mice. Cancer Immunol Res (2015) 3(4):333–44. 10.1158/2326-6066.CIR-14-0114 25492700

[52] Muller T Uherek C Maki G Chow KU Schimpf A Klingemann HG Expression of a CD20-specific chimeric antigen receptor enhances cytotoxic activity of NK cells and overcomes NK-resistance of lymphoma and leukemia cells. Cancer Immunol Immunother (2008) 57(3):411–23. 10.1007/s00262-007-0383-3 PMC11029838 17717662

[53] Jiang H Zhang W Shang P Zhang H Fu W Ye F Transfection of chimeric anti-CD138 gene enhances natural killer cell activation and killing of multiple myeloma cells. Mol Oncol (2014) 8(2):297–310. 10.1016/j.molonc.2013.12.001 24388357 PMC5528539

[54] Chen KH Wada M Firor AE Pinz KG Jares A Liu H Novel anti-CD3 chimeric antigen receptor targeting of aggressive T cell malignancies. Oncotarget (2016) 7(35):56219–32. 10.18632/oncotarget.11019 PMC5302909 27494836

[55] Chen KH Wada M Pinz KG Liu H Lin KW Jares A Preclinical targeting of aggressive T-cell malignancies using anti-CD5 chimeric antigen receptor. Leukemia (2017) 31(10):2151–60. 10.1038/leu.2017.8 PMC5629371 28074066

[56] McLellan AD Ali Hosseini Rad SM Chimeric antigen receptor T cell persistence and memory cell formation. Immunol Cell Biol (2019) 97(7):664–74. 10.1111/imcb.12254 31009109

[57] Netea MG Latz E Mills KH O’Neill LA Innate immune memory: a paradigm shift in understanding host defense. Nat Immunol (2015) 16(7):675–9. 10.1038/ni.3178 26086132

[58] Mukherjee S Jensen H Stewart W Stewart D Ray WC Chen SY In silico modeling identifies CD45 as a regulator of IL-2 synergy in the NKG2D-mediated activation of immature human NK cells. Sci Signal (2017) 10(485):eaai9062. 10.1126/scisignal.aai9062 28655861 PMC5556952

[59] Conlon KC Lugli E Welles HC Rosenberg SA Fojo AT Morris JC Redistribution, hyperproliferation, activation of natural killer cells and CD8 T cells, and cytokine production during first-in-human clinical trial of recombinant human interleukin-15 in patients with cancer. J Clin Oncol (2015) 33(1):74–82. 10.1200/JCO.2014.57.3329 25403209 PMC4268254

[60] Romee R Schneider SE Leong JW Chase JM Keppel CR Sullivan RP Cytokine activation induces human memory-like NK cells. Blood (2012) 120(24):4751–60. 10.1182/blood-2012-04-419283 PMC3520618 22983442

[61] Lee J Zhang T Hwang I Kim A Nitschke L Kim M Epigenetic modification and antibody-dependent expansion of memory-like NK cells in human cytomegalovirus-infected individuals. Immunity (2015) 42(3):431–42. 10.1016/j.immuni.2015.02.013 PMC4537797 25786175

[62] Gang M Marin ND Wong P Neal CC Marsala L Foster M CAR-modified memory-like NK cells exhibit potent responses to NK-resistant lymphomas. Blood (2020) 9:200–18. 10.1182/blood.2020006619 PMC7702478 32614951

[63] Shimasaki N Jain A Campana D NK cells for cancer immunotherapy. Nat Rev Drug Discov (2020) 19(3):200–18. 10.1038/s41573-019-0052-1 31907401

[64] Makanga DR Lorenzo FDRD David G Willem C Dubreuil L Legrand N Genetic and Molecular Basis of Heterogeneous NK Cell Responses against Acute Leukemia Cancers. Cancers (2020) 12(7):1927–44. 10.3390/cancers12071927 PMC7409189 32708751

[65] Tonn T Schwabe D Klingemann HG Becker S Esser R Koehl U Treatment of patients with advanced cancer with the natural killer cell line NK-92. Cytotherapy (2013) 15(12):1563–70. 10.1016/j.jcyt.2013.06.017 24094496

[66] Ingegnere T Mariotti FR Pelosi A Quintarelli C De Angelis B Tumino N Human CAR NK Cells: A New Non-viral Method Allowing High Efficient Transfection and Strong Tumor Cell Killing. Front Immunol (2019) 10:957. 10.3389/fimmu.2019.00957 31114587 PMC6503170

[67] Liu E Tong Y Dotti G Shaim H Savoldo B Mukherjee M Cord blood NK cells engineered to express IL-15 and a CD19-targeted CAR show long-term persistence and potent antitumor activity. Leukemia (2018) 32(2):520–31. 10.1038/leu.2017.226 PMC6063081 28725044

[68] Sharma R Das A IL-2 mediates NK cell proliferation but not hyperactivity. Immunol Res (2018) 66(1):151–7. 10.1007/s12026-017-8982-3 29256180

[69] Hannani D Ma Y Yamazaki T Dechanet-Merville J Kroemer G Zitvogel L Harnessing gammadelta T cells in anticancer immunotherapy. Trends Immunol (2012) 33(5):199–206. 10.1016/j.it.2012.01.006 22364810

[70] Legut M Cole DK Sewell AK The promise of gammadelta T cells and the gammadelta T cell receptor for cancer immunotherapy. Cell Mol Immunol (2015) 12(6):656–68. 10.1038/cmi.2015.28 PMC4716630 25864915

[71] Gentles AJ Newman AM Liu CL Bratman SV Feng W Kim D The prognostic landscape of genes and infiltrating immune cells across human cancers. Nat Med (2015) 21(8):938–45. 10.1038/nm.3909 PMC4852857 26193342

[72] Tosolini M Pont F Poupot M Vergez F Nicolau-Travers ML Vermijlen D Assessment of tumor-infiltrating TCRVgamma9Vdelta2 gammadelta lymphocyte abundance by deconvolution of human cancers microarrays. Oncoimmunology (2017) 6(3):e1284723. 10.1080/2162402X.2017.1284723 28405516 PMC5384348

[73] Rischer M Pscherer S Duwe S Vormoor J Jurgens H Rossig C Human gammadelta T cells as mediators of chimaeric-receptor redirected anti-tumour immunity. Br J Haematol (2004) 126(4):583–92. 10.1111/j.1365-2141.2004.05077.x 15287953

[74] Zoine JT Knight KA Fleischer LC Sutton KS Goldsmith KC Doering CB Ex vivo expanded patient-derived gammadelta T-cell immunotherapy enhances neuroblastoma tumor regression in a murine model. Oncoimmunology (2019) 8(8):1593804. 10.1080/2162402X.2019.1593804 31413905 PMC6682349

[75] Ghassemi S Nunez-Cruz S O’Connor RS Fraietta JA Patel PR Scholler J Reducing Ex Vivo Culture Improves the Antileukemic Activity of Chimeric Antigen Receptor (CAR) T Cells. Cancer Immunol Res (2018) 6(9):1100–9. 10.1158/2326-6066.CIR-17-0405 PMC8274631 30030295

[76] Kunzmann V Bauer E Feurle J Weissinger F Tony HP Wilhelm M Stimulation of gammadelta T cells by aminobisphosphonates and induction of antiplasma cell activity in multiple myeloma. Blood (2000) 96(2):384–92. 10.1182/blood.V96.2.384 10887096

[77] Gu S Nawrocka W Adams EJ Sensing of Pyrophosphate Metabolites by Vgamma9Vdelta2 T Cells. Front Immunol (2014) 5:688. 10.3389/fimmu.2014.00688 25657647 PMC4303140

[78] Compte E Pontarotti P Collette Y Lopez M Olive D Frontline: Characterization of BT3 molecules belonging to the B7 family expressed on immune cells. Eur J Immunol (2004) 34(8):2089–99. 10.1002/eji.200425227 15259006

[79] Zocchi MR Costa D Vene R Tosetti F Ferrari N Minghelli S Zoledronate can induce colorectal cancer microenvironment expressing BTN3A1 to stimulate effector gammadelta T cells with antitumor activity. Oncoimmunology (2017) 6(3):e1278099. 10.1080/2162402X.2016.1278099 28405500 PMC5384426

[80] Le Page C Marineau A Bonza PK Rahimi K Cyr L Labouba I BTN3A2 expression in epithelial ovarian cancer is associated with higher tumor infiltrating T cells and a better prognosis. PLoS One (2012) 7(6):e38541. 10.1371/journal.pone.0038541 22685580 PMC3369854

[81] Blazquez JL Benyamine A Pasero C Olive D New Insights Into the Regulation of gammadelta T Cells by BTN3A and Other BTN/BTNL in Tumor Immunity. Front Immunol (2018) 9:1601. 10.3389/fimmu.2018.01601 30050536 PMC6050389

[82] Brudno JN Somerville RP Shi V Rose JJ Halverson DC Fowler DH Allogeneic T Cells That Express an Anti-CD19 Chimeric Antigen Receptor Induce Remissions of B-Cell Malignancies That Progress After Allogeneic Hematopoietic Stem-Cell Transplantation Without Causing Graft-Versus-Host Disease. J Clin Oncol (2016) 34(10):1112–21. 10.1200/JCO.2015.64.5929 PMC4872017 26811520

[83] Kolb HJ Graft-versus-leukemia effects of transplantation and donor lymphocytes. Blood (2008) 112(12):4371–83. 10.1182/blood-2008-03-077974 19029455

[84] Frey NV Porter DL Graft-versus-host disease after donor leukocyte infusions: presentation and management. Best Pract Res Clin Haematol (2008) 21(2):205–22. 10.1016/j.beha.2008.02.007 PMC2504712 18503987

[85] Roddie C Peggs KS Donor lymphocyte infusion following allogeneic hematopoietic stem cell transplantation. Expert Opin Biol Ther (2011) 11(4):473–87. 10.1517/14712598.2011.554811 21269237

[86] Mackinnon S Papadopoulos EB Carabasi MH Reich L Collins NH Boulad F Adoptive immunotherapy evaluating escalating doses of donor leukocytes for relapse of chronic myeloid leukemia after bone marrow transplantation: separation of graft-versus-leukemia responses from graft-versus-host disease. Blood (1995) 86(4):1261–8. 10.1182/blood.V86.4.1261.bloodjournal8641261 7632930

[87] Cruz CR Micklethwaite KP Savoldo B Ramos CA Lam S Ku S Infusion of donor-derived CD19-redirected virus-specific T cells for B-cell malignancies relapsed after allogeneic stem cell transplant: a phase 1 study. Blood (2013) 122(17):2965–73. 10.1182/blood-2013-06-506741 PMC3811171 24030379

[88] Kochenderfer JN Dudley ME Carpenter RO Kassim SH Rose JJ Telford WG Donor-derived CD19-targeted T cells cause regression of malignancy persisting after allogeneic hematopoietic stem cell transplantation. Blood (2013) 122(25):4129–39. 10.1182/blood-2013-08-519413 PMC3862276 24055823

[89] Yang Y Hu Y Huang H Ruxolitinib treatment for acute gastrointestinal graft-versus-host disease caused by donor-derived CD19-Chimeric antigen receptor T-Cell infusion in a patient with B-ALL relapsed after Allo-HSCT. Regener Ther (2019) 11:139–42. 10.1016/j.reth.2019.06.006 PMC6646647 31367654

[90] Qiu H Allogeneic Donor-Derived Anti-CD19 CAR T Cell Is a Promising Therapy for Relapsed/Refractory B-ALL After Allogeneic Hematopoietic Stem-Cell Transplantation. Clin Lymphoma Myeloma Leukemia (2020) 20: 610–6. 10.1016/j.clml.2020.04.007 32507386

[91] Wiebking V Lee CM Mostrel N Lahiri P Bak R Bao G Genome editing of donor-derived T-cells to generate allogenic chimeric antigen receptor-modified T cells: Optimizing alphabeta T cell-depleted haploidentical hematopoietic stem cell transplantation. Haematologica (2020) epub ahead of print. 10.3324/haematol.2019.233882 PMC7928014 32241852

[92] Rocha V Gluckman E Eurocord-Netcord R European B Marrow Transplant g. Improving outcomes of cord blood transplantation: HLA matching, cell dose and other graft- and transplantation-related factors. Br J Haematol (2009) 147(2):262–74. 10.1111/j.1365-2141.2009.07883.x 19796275

[93] Eapen M Rocha V Sanz G Scaradavou A Zhang MJ Arcese W Effect of graft source on unrelated donor haemopoietic stem-cell transplantation in adults with acute leukaemia: a retrospective analysis. Lancet Oncol (2010) 11(7):653–60. 10.1016/S1470-2045(10)70127-3 PMC3163510 20558104

[94] Kwoczek J Riese SB Tischer S Bak S Lahrberg J Oelke M Cord blood-derived T cells allow the generation of a more naive tumor-reactive cytotoxic T-cell phenotype. Transfusion (2018) 58(1):88–99. 10.1111/trf.14365 29023759

[95] Kadereit S Mohammad SF Miller RE Woods KD Listrom CD McKinnon K Reduced NFAT1 protein expression in human umbilical cord blood T lymphocytes. Blood (1999) 94(9):3101–7. 10.1182/blood.V94.9.3101.421k04_3101_3107 10556195

[96] Rocha V Cornish J Sievers EL Filipovich A Locatelli F Peters C Comparison of outcomes of unrelated bone marrow and umbilical cord blood transplants in children with acute leukemia. Blood (2001) 97(10):2962–71. 10.1182/blood.V97.10.2962 11342418

[97] Serrano LM Pfeiffer T Olivares S Numbenjapon T Bennitt J Kim D Differentiation of naive cord-blood T cells into CD19-specific cytolytic effectors for posttransplantation adoptive immunotherapy. Blood (2006) 107(7):2643–52. 10.1182/blood-2005-09-3904 PMC1895371 16352804

[98] Grupp SA Kalos M Barrett D Aplenc R Porter DL Rheingold SR Chimeric antigen receptor-modified T cells for acute lymphoid leukemia. N Engl J Med (2013) 368(16):1509–18. 10.1056/NEJMoa1215134 PMC4058440 23527958

[99] Lund TC Boitano AE Delaney CS Shpall EJ Wagner JE Advances in umbilical cord blood manipulation-from niche to bedside. Nat Rev Clin Oncol (2015) 12(3):163–74. 10.1038/nrclinonc.2014.215 PMC4430198 25511187

[100] Pegram HJ Purdon TJ van Leeuwen DG Curran KJ Giralt SA Barker JN IL-12-secreting CD19-targeted cord blood-derived T cells for the immunotherapy of B-cell acute lymphoblastic leukemia. Leukemia (2015) 29(2):415–22. 10.1038/leu.2014.215 PMC5189717 25005243

[101] Juch H Blaschitz A Dohr G Hutter H HLA class I expression in the human placenta. Wien Med Wochenschr (2012) 162(9-10):196–200. 10.1007/s10354-012-0070-7 22717873

[102] Montel-Hagen A Seet CS Li S Chick B Zhu Y Chang P Organoid-Induced Differentiation of Conventional T Cells from Human Pluripotent Stem Cells. Cell Stem Cell (2019) 24(3):376–89.e8. 10.1016/j.stem.2018.12.011 30661959 PMC6687310

[103] Sheldon S Poulton K HLA typing and its influence on organ transplantation. Methods Mol Biol (2006) 333:157–74. 10.1385/1-59745-049-9:157 16790851

[104] Lim WH Chapman JR Coates PT Lewis JR Russ GR Watson N HLA-DQ Mismatches and Rejection in Kidney Transplant Recipients. Clin J Am Soc Nephrol (2016) 11(5):875–83. 10.2215/CJN.11641115 PMC4858494 27034399

[105] Taylor CJ Peacock S Chaudhry AN Bradley JA Bolton EM Generating an iPSC bank for HLA-matched tissue transplantation based on known donor and recipient HLA types. Cell Stem Cell (2012) 11(2):147–52. 10.1016/j.stem.2012.07.014 22862941

[106] Krawczyk M Peyraud N Rybtsova N Masternak K Bucher P Barras E Long distance control of MHC class II expression by multiple distal enhancers regulated by regulatory factor X complex and CIITA. J Immunol (2004) 173(10):6200–10. 10.4049/jimmunol.173.10.6200 15528357

[107] Torikai H Reik A Soldner F Warren EH Yuen C Zhou Y Toward eliminating HLA class I expression to generate universal cells from allogeneic donors. Blood (2013) 122(8):1341–9. 10.1182/blood-2013-03-478255 PMC3750336 23741009

[108] Choi BD Yu X Castano AP Darr H Henderson DB Bouffard AA CRISPR-Cas9 disruption of PD-1 enhances activity of universal EGFRvIII CAR T cells in a preclinical model of human glioblastoma. J Immunother Cancer (2019) 7(1):304. 10.1186/s40425-019-0806-7 31727131 PMC6857271

[109] Gornalusse GG Hirata RK Funk SE Riolobos L Lopes VS Manske G HLA-E-expressing pluripotent stem cells escape allogeneic responses and lysis by NK cells. Nat Biotechnol (2017) 35(8):765–72. 10.1038/nbt.3860 PMC5548598 28504668

[110] Jandus C Boligan KF Chijioke O Liu H Dahlhaus M Demoulins T Interactions between Siglec-7/9 receptors and ligands influence NK cell-dependent tumor immunosurveillance. J Clin Invest (2014) 124(4):1810–20. 10.1172/JCI65899 PMC3973073 24569453

[111] Mo F Watanabe N McKenna MK Hicks MJ Srinivasan M Gomes-Silva D Engineered off-the-shelf therapeutic T cells resist host immune rejection. Nat Biotechnol (2020) online ahead of print. 10.1038/s41587-020-0601-5 PMC7854790 32661440

[112] Urbanska K Lanitis E Poussin M Lynn RC Gavin BP Kelderman S A universal strategy for adoptive immunotherapy of cancer through use of a novel T-cell antigen receptor. Cancer Res (2012) 72(7):1844–52. 10.1158/0008-5472.CAN-11-3890 PMC3319867 22315351

[113] Davila ML Bouhassira DC Park JH Curran KJ Smith EL Pegram HJ Chimeric antigen receptors for the adoptive T cell therapy of hematologic malignancies. Int J Hematol (2014) 99(4):361–71. 10.1007/s12185-013-1479-5 PMC4684946 24311149

[114] Lohmueller JJ Ham JD Kvorjak M Finn OJ mSA2 affinity-enhanced biotin-binding CAR T cells for universal tumor targeting. Oncoimmunology (2017) 7(1):e1368604. 10.1080/2162402X.2017.1368604 29296519 PMC5739565

[115] Kudo K Imai C Lorenzini P Kamiya T Kono K Davidoff AM T lymphocytes expressing a CD16 signaling receptor exert antibody-dependent cancer cell killing. Cancer Res (2014) 74(1):93–103. 10.1158/0008-5472.CAN-13-1365 24197131

[116] Rodgers DT Mazagova M Hampton EN Cao Y Ramadoss NS Hardy IR Switch-mediated activation and retargeting of CAR-T cells for B-cell malignancies. Proc Natl Acad Sci U S A (2016) 113(4):E459–68. 10.1073/pnas.1524155113 PMC4743815 26759369

[117] Cho JH Collins JJ Wong WW Universal Chimeric Antigen Receptors for Multiplexed and Logical Control of T Cell Responses. Cell (2018) 173(6):1426– 10.1016/j.cell.2018.03.038 29706540 PMC5984158

[118] Reusch D Tejada ML Fc glycans of therapeutic antibodies as critical quality attributes. Glycobiology (2015) 25(12):1325–34. 10.1093/glycob/cwv065 PMC4634315 26263923

[119] Walsh S Shah A Mond J Improved pharmacokinetics and reduced antibody reactivity of lysostaphin conjugated to polyethylene glycol. Antimicrob Agents Chemother (2003) 47(2):554–8. 10.1128/AAC.47.2.554-558.2003 PMC151727 12543658

[120] Stratton SL Horvath TD Bogusiewicz A Matthews NI Henrich CL Spencer HJ Plasma concentration of 3-hydroxyisovaleryl carnitine is an early and sensitive indicator of marginal biotin deficiency in humans. Am J Clin Nutr (2010) 92(6):1399–405. 10.3945/ajcn.110.002543 PMC2980966 20943794

[121] Bai S Jorga K Xin Y Jin D Zheng Y Damico-Beyer LA A guide to rational dosing of monoclonal antibodies. Clin Pharmacokinet (2012) 51(2):119–35. 10.2165/11596370-000000000-00000 22257150

[122] Mohning MP Downey GP Cosgrove GP Redente EF “Chapter 3 - Mechanisms of Fibrosis”. In: Swigris JJ Brown KK, editors. Idiopathic Pulmonary Fibrosis. (2019). Elsevier p. 9–31 10.1016/B978-0-323-54431-3.00003-2

[123] Qasim W Ciocarlie O Adams S Inglott S Murphy C Rivat C Preliminary Results of UCART19, an Allogeneic Anti-CD19 CAR T-Cell Product in a First-in-Human Trial (PALL) in Pediatric Patients with CD19+ Relapsed/Refractory B-Cell Acute Lymphoblastic Leukemia. Blood (2017) 130(Supplement 1):1271. 10.1182/blood.V130.Suppl_1.887.887

[124] Graham C Yallop D Jozwik A Patten P Dunlop A Ellard R Preliminary Results of UCART19, an Allogeneic Anti-CD19 CAR T-Cell Product, in a First-in-Human Trial (CALM) in Adult Patients with CD19+ Relapsed/Refractory B-Cell Acute Lymphoblastic Leukemia. Blood (2017) 130(Supplement 1):887– 10.1182/blood.V130.Suppl_1.887.887

[125] Guzman ML Sugita M Zong H Ewing-Crystal N Trujillo-Alonso V Mencia-Trinchant N Allogeneic Tcrα/β Deficient CAR T-Cells Targeting CD123 Prolong Overall Survival of AML Patient-Derived Xenografts. Blood (2016) 128(22):765–. 10.1182/blood.V128.22.765.765

[126] Cai T Galetto R Gouble A Smith J Cavazos A Konoplev S Pre-Clinical Studies of Anti-CD123 CAR-T Cells for the Treatment of Blastic Plasmacytoid Dendritic Cell Neoplasm (BPDCN). Blood (2016) 128(22):4039–. 10.1182/blood.V128.22.4039.4039

[127] Cellectis Cellectis reports clinical hold of UCART123 studies 2017. (2017) (New York: Cellectis) Available at: http://www.cellectis.com/en/press/cellectis-reports-clinical-hold-of-ucart123-studies.

[128] Mardiros A Forman SJ Budde LE T cells expressing CD123 chimeric antigen receptors for treatment of acute myeloid leukemia. Curr Opin Hematol (2015) 22(6):484–8. 10.1097/MOH.0000000000000190 PMC4624420 26457961

[129] Fleming JN Nash RA McLeod DO Fiorentino DF Shulman HM Connolly MK Capillary regeneration in scleroderma: stem cell therapy reverses phenotype? PLoS One (2008) 3(1):e1452. 10.1371/journal.pone.0001452 18197262 PMC2175530

[130] Therapeutics C Creating transformative gene-based medicines for serious diseases: corporate overview. (CRISPR THERAPEUTICS company) (2020). Available at: https://crisprtx.gcs-web.com/static-files/6a4a2db8-faf2-4fe9-a2b7-d81a05312e9f.

[131] Lee DW Gardner R Porter DL Louis CU Ahmed N Jensen M Current concepts in the diagnosis and management of cytokine release syndrome. Blood (2014) 124(2):188–95. 10.1182/blood-2014-05-552729 PMC4093680 24876563

[132] Peppa D Micco L Javaid A Kennedy PT Schurich A Dunn C Blockade of immunosuppressive cytokines restores NK cell antiviral function in chronic hepatitis B virus infection. PLoS Pathog (2010) 6(12):e1001227. 10.1371/journal.ppat.1001227 21187913 PMC3003000

[133] Nimer SD Uchida H Regulation of granulocyte-macrophage colony-stimulating factor and interleukin 3 expression. Stem Cells (1995) 13(4):324–35. 10.1002/stem.5530130402 7549890

[134] Tang X Yang L Li Z Nalin AP Dai H Xu T Erratum: First-in-man clinical trial of CAR NK-92 cells: safety test of CD33-CAR NK-92 cells in patients with relapsed and refractory acute myeloid leukemia. Am J Cancer Res (2018) 8(9):1899. 30323981 PMC6176185

[135] Koch J Steinle A Watzl C Mandelboim O Activating natural cytotoxicity receptors of natural killer cells in cancer and infection. Trends Immunol (2013) 34(4):182–91. 10.1016/j.it.2013.01.003 23414611

[136] Alderson KL Sondel PM Clinical cancer therapy by NK cells via antibody-dependent cell-mediated cytotoxicity. J BioMed Biotechnol (2011) 2011:379123. 10.1155/2011/379123 21660134 PMC3110303

[137] Tal Y Yaakobi S Horovitz-Fried M Safyon E Rosental B Porgador A An NCR1-based chimeric receptor endows T-cells with multiple anti-tumor specificities. Oncotarget (2014) 5(21):10949–58. 10.18632/oncotarget.1919 PMC4279421 25431955

[138] Barber A Rynda A Sentman CL Chimeric NKG2D expressing T cells eliminate immunosuppression and activate immunity within the ovarian tumor microenvironment. J Immunol (2009) 183(11):6939–47. 10.4049/jimmunol.0902000 PMC2825039 19915047

[139] Zhang T Sentman CL Cancer immunotherapy using a bispecific NK receptor fusion protein that engages both T cells and tumor cells. Cancer Res (2011) 71(6):2066–76. 10.1158/0008-5472.CAN-10-3200 PMC3095211 21282338

[140] Tasian SK Kenderian SS Shen F Ruella M Shestova O Kozlowski M Optimized depletion of chimeric antigen receptor T cells in murine xenograft models of human acute myeloid leukemia. Blood (2017) 129(17):2395–407. 10.1182/blood-2016-08-736041 PMC5409446 28246194

[141] Klichinsky M Ruella M Shestova O Lu XM Best A Zeeman M Human chimeric antigen receptor macrophages for cancer immunotherapy. Nat Biotechnol (2020) 38(8):947–53. 10.1038/s41587-020-0462-y PMC7883632 32361713

[142] Mirzaei HR Rodriguez A Shepphird J Brown CE Badie B Chimeric Antigen Receptors T Cell Therapy in Solid Tumor: Challenges and Clinical Applications. Front Immunol (2017) 8:1850. 10.3389/fimmu.2017.01850 29312333 PMC5744011

[143] Zhang E Gu J Xu H Prospects for chimeric antigen receptor-modified T cell therapy for solid tumors. Mol Cancer (2018) 17(1):7. 10.1186/s12943-018-0759-3 29329591 PMC5767005

[144] Castellarin M Watanabe K June CH Kloss CC Posey AD Jr. Driving cars to the clinic for solid tumors. Gene Ther (2018) 25(3):165–75. 10.1038/s41434-018-0007-x 29880908

[145] D’Aloia MM Zizzari IG Sacchetti B Pierelli L Alimandi M CAR-T cells: the long and winding road to solid tumors. Cell Death Dis (2018) 9(3):282. 10.1038/s41419-018-0278-6 29449531 PMC5833816

